# Biomass and Bioenergy Potential of Brown Midrib Sweet Sorghum Germplasm

**DOI:** 10.3389/fpls.2019.01142

**Published:** 2019-09-20

**Authors:** Luis A. Rivera-Burgos, Jeffrey J. Volenec, Gebisa Ejeta

**Affiliations:** Department of Agronomy, Purdue University, West Lafayette, IN, United States

**Keywords:** *Sorghum bicolor*, recombinant inbred lines, lignocellulosic biomass, brown midrib mutation, stem sugar concentration, ethanol

## Abstract

Public appetite for fossil fuels continues to drive energy prices and foment the build-up of intractable environmental problems. Ethanol (ETOH) production from lignocellulosic biomass grown in marginal lands offers a sustainable alternative without diverting arable land from food and feed production. The quantity and quality of lignocellulosic biomass can be enhanced by the abundant genetic diversity for biomass production as well as stem sugar and lignin composition in sorghum (*Sorghum bicolor* L. Moench). The objective of this study was to assess yield and quality of lignocellulosic biomass enhancement for ethanol production potential in a population of sorghum derived from two cultivars with contrasting biomass yield and compositional traits. We tested 236 recombinant inbred lines (RIL) of sorghum in a randomized complete block design (RCBD) with two replications for lignocellulosic biomass performance and determined hemicellulose, cellulose and lignin concentrations through detergent fiber analysis (DFA). The stover compositional values were used to estimate theoretical ethanol yield (ETOH on a mass basis) and production (ETOH on an area basis). Results showed that RIL carrying the brown midrib mutation had significantly higher theoretical glucose recovery (released glucose from cellulose, > 200 g kg^-1^). Those carrying both mutations, had high theoretical ethanol yield (>400 L ton^-1^) and high theoretical ethanol production (>14,500 L ha^-1^). Lignin concentration was determined as most reliable predictor (R^2^ = 0.67) for glucose recovery. Lignin and stem sugar concentrations (R^2^ = 0.46 and 0.35, respectively) were good predictors for ethanol yield. Stover yield traits (R^2^ = 0.89) were most important determinants for ethanol production. Our findings suggest that careful breeding of sorghum for genetic enhancement of biomass quantity and quality could double lignocellulosic ethanol yields.

## Introduction

Fossil fuel dependency and increased greenhouse gases are major concerns that have caught the attention of environmentalists, economists, as well as scientists. Fossil fuel is a non-renewable source of energy, with its production and utilization generating gases, associated with environmental pollution as well as causing respiratory health problems. However, rapid expansion of the global economy sustains an insatiable hunger for energy, leading to a strong dependency on fossil fuel products. This dependency creates a growing consumer demand that raises petroleum product prices affecting the economy within and among countries (Davis et al., 2008).

A large volume of lignocellulosic biomass is produced from a number of crops every year. Field crops produce considerable amounts of both grain and stover. Grain from crops is harvested and used for human (food) and animal (feed) consumption, while stover (lignocellulosic biomass) is often left unharvested on-farm every crop season (Nelson et al., 2011). Though some may consider the stover left on-farm a waste, agronomists recognize the value of crop residues for reducing soil erosion and building soil organic matter (Gupta and Verma, 2015).

Sorghum research conducted over the last several years has generated interest in this stress-tolerant species as a potential biomass crop for lignocellulosic feedstock and energy production (Rooney et al., 2007). Knowledge on the genetics of several lignocellulosic traits in sorghum is also growing, albeit slowly due to a constrained research funding environment. The sweet-stalk phenotype appears to be a quantitatively inherited trait, controlled by multiple loci. Recent genetic analyses have placed quantitative trait loci (QTL) for stem sugar concentration on four chromosomes (3, 5, 6, and 7). These QTL generally explain from 11% to 21% of the total variation for stem sugar concentration (Ritter et al., 2008; Murray et al., 2008a; Murray et al., 2009). It is suggested that environmental factors as well as additional unidentified QTL likely affect the expression of this trait.

Brown midrib mutants have been identified in maize (*Zea mays*) and sorghum (*Sorghum bicolor*) by either spontaneous or chemical mutagenesis. Five brown midrib (*bm1* through *bm5*) loci have been identified, extensively studied, and genetically characterized in maize. The brown midrib phenotype (reddish-brown coloration) is correlated with two homologous loci in maize (*bm1* and *bm3*) and sorghum (*bmr6* and *bmr12*). In both grasses, the brow midrib phenotype is associated with reduced lignin concentration and increased livestock digestibility (Sattler et al., 2010).

The brown-midrib (*bmr*) mutation in sorghum, caused by single-point mutations in genes involved in cell wall composition, generally reduces lignin concentration. The *bmr* mutants are visually identified by the reddish-brown coloration in the midrib of sorghum leaves that is associated with low lignin concentration. Several *bmr* mutant lines were identified at Purdue University from a chemical mutagenesis study aimed at improving sorghum forage quality (Porter et al., 1978). The *bmr* mutant collection was tested for allelism and they fall into four *bmr* groups, all causing reduced lignin concentration in sorghum lignocellulosic biomass (Saballos et al., 2008). Group 1 mutants include *bmr3*, *bmr4*, *bmr6*, *bmr27*, and *bmr28*; Group 2 mutants contain *bmr7*, *bmr12*, *bmr18*, *bmr25*, and *bmr26*; Group 3 mutants include *bmr19* only; and allelic Group 4 includes *bmr2*, *bmr5*, and *bmr14*. From these groups, two genes have been identified to be involved in the lignin biosynthetic pathway. One identified gene that belongs to allelic group 1, is located on chromosome 3, and affects cinnamyl alcohol dehydrogenase (CAD) activity during lignin biosynthesis. The CAD is encoded by a multi-gene family consisting of members thought to have distinct roles (Palmer et al., 2008; Saballos et al., 2008). Another locus on chromosome 7, belonging to allelic group 2, is responsible for low activity of the enzyme caffeic acid o-methyltransferase (COMT) (Bout and Vermerris, 2003). This enzyme also plays an important role during lignin biosynthesis in sorghum.

Stover lignin concentration plays an important role during enzymatic hydrolysis of cellulose. During this process, increased amount of lignin prevents the attachment of the hydrolytic enzyme to cellulose, and leads to a low yield of fermentable sugars. Therefore, high concentration of lignin in stover could lead to low ethanol yields (Sun and Cheng, 2002; Ohgren et al., 2007).

Traditionally, commercial lignocellulosic ethanol production is based on conversion of non-structural carbohydrates (soluble carbohydrates) or structural carbohydrates (hemicellulose and cellulose) to ethanol. Soluble carbohydrates (sucrose, glucose, and fructose) are generally referred to fermentable sugars and these accumulate in stems of crops like sugarcane (*Saccharum officinarum*) and sweet sorghums (Han et al., 2013). Due to the simple biochemical structure of these fermentable sugars, they can be converted to ethanol in a single step known as simultaneous saccharification-fermentation (SSF) (Saballos et al., 2008).

Structural carbohydrates form part of the plant cell wall and are tightly linked to lignin. During biomass conversion, these complex sugars undergo three different processes to produce ethanol as their final product. The first process separates lignin from the complex carbohydrates using pretreatment of hot sulfuric acid (Dien et al., 2006). The second process hydrolyzes the complex carbohydrates (cellulose and hemicellulose) to simple carbohydrates and the third process ferments simple carbohydrates to ethanol (Sun and Cheng, 2002; Dien et al., 2006; Sticklen, 2008; Canilha et al., 2012). These processes suggest that the combination of both sources of carbohydrates in one crop is highly desirable to increase ethanol production from lignocellulosic biomass (Rooney et al., 2007).

A genetically enhanced biomass that combines both sources of carbohydrates, maximizing the soluble carbohydrate pool, but also making the structural carbohydrates of cell walls readily fermentable, increases the value of a feedstock (Oliver et al., 2005; Vogler et al., 2009; Gírio et al., 2010; Vogel et al., 2011). We hypothesize that the combination of two compositional mutations segregating in brown × sweet sorghum recombinant inbred lines (RIL) would increase the fermentable carbohydrate pool of feedstock. The objectives of this study were: 1) To assess biomass component traits of *bmr* x sweet sorghum RIL; 2) To determine the concentration of cellulose, hemicellulose and lignin (compositional traits) of *bmr* × sweet sorghum RIL; 3) To estimate theoretical glucose recovery, theoretical ethanol yield, and theoretical ethanol production from two sources of carbohydrates; and 4) To determine suitable predictors associated with theoretical ethanol.

## Materials and Methods

### Plant Material

A population consisting of 236 RIL was evaluated in a 2-year study. The population was developed through seven generations of single-seed descent selection from the original F_2_ population of a cross between two lines, *bmr12* (a brown midrib, low lignin sorghum) and Brown County (a sweet sorghum) as parents. Additionally, a forage sorghum cultivar (*bmr*Atlas) with an introgressed *bmr 12 gene* was included as control.

### Field Experiment

The experiment was planted in late May at the Agronomy Center for Research and Education (ACRE) in West Lafayette, Indiana (40°29’41.67”N; 86°59’26.46”W). The soils at ACRE consist of Drummer silty clay loam (fine-silty, mixed, superactive, mesic Typic Endoaquolls) and Raub silt loam (fine-silty, mixed, superactive, mesic Aquic Argiudolls) (United States Department of Agriculture, 2014). Mean air temperature and precipitation at ACRE during the crop seasons of 2008 and 2009 are shown in **Table 1**. Rainfall totals for the growing season (May–October) in 2008 and 2009 were 586.0 and 631 mm, respectively. Monthly mean temperatures (average monthly minimum and maximum) for the growing season for each year were 18.3ºC (3.3–28.5 ºC) in 2008 and 18.0ºC (4.0–28.0ºC) in 2010 (Indiana State Climate Office, 2014).

**Table 1:** Mean air temperature and precipitation at Lafayette IN during the crop season of 2008 and 2009.

	Precipitation		Avg. mean temp		Avg. min temp		Avg. max temp	
	(in)	mm	(°F)	(ºC)	(°F)	(ºC)	(°F)	(ºC)
2008								
May	6.0	151	57	14	46	8	68	20
June	4.9	124	72	22	62	17	83	28
July	3.8	97	73	23	62	17	83	29
August	2.4	61	69	21	57	14	82	28
September	4.2	108	66	19	53	12	80	27
October	1.8	45	52	11	38	3	66	19
Average	4	98	65	18	53	12	77	25
2009								
May	5.2	132	62	17	50	10	74	23
June	5.7	146	72	22	62	16	82	28
July	3.0	77	69	20	59	15	79	26
August	4.2	107	70	21	59	15	81	27
September	0.6	14	64	18	52	11	76	25
October	6.1	155	50	10	40	4	59	15
Average	4	105	64	18	53	12	75	24

All RIL, both parents, and *bmr*Atlas were each planted in two-row plots. Dimensions of each plot were 6.10 m long with 0.76 m spacing between rows. Approximately 2.5 grams sorghum seed row^-1^ was planted at a depth of 5 cm. The seeds were treated with a fungicide prior to planting to ensure better seedling emergence and stand establishment. Three weeks after planting, plots were thinned to 6 plants per 30 cm for an approximate plant population of 250,000 plants per hectare. Urea ammonium nitrate was applied and incorporated at a rate of 150 kg N per hectare.

### Biomass and Stem Sugar Measurements

Plant maturity (PM) of grain was defined as 45 days after flowering date. Based on flowering dates, RIL were placed into three PM groups in order to minimize confounding differences of PM in biomass component and compositional traits. Stem sugar concentrations (SSC) were estimated as degrees Brix (ºBrix) of each RIL, parents and commercial control at PM. For each plot, four plants located in the middle region of each row were randomly collected and stem internodes between the fourth and the fifth node squeezed with a garlic press to obtain the stem juice. A digital refractometer (ATAGO Model PAL-1) was used to measure Brix (One degree Brix is 1 g of soluble sugars in 100 g of solution) (Han et al., 2013). The average of the four brix measurements per plot was used to determine soluble carbohydrates. At harvesting time of each PM group (Stage 9), a sample plot of 10 plants (5 from each row) was randomly selected from the middle region of each plot. Panicles were removed, and the weight of leaves and stems was recorded as fresh stover yield (FSY) per plot. These 10 plants were passed through a tractor-powered mechanical chopper, chopped leaves and stems mixed, and a subsample of 300 g was weighed, dried in a forced-air dryer for 3–4 days at 60ºC, and dry stover subsample weight was recorded. Dry stover yield (DSY) per plot was calculated by dividing the dry stover subsamples weight by fresh stover subsample weight and multiplying by FSY per plot (Murray et al., 2008b). Dry stover subsamples were ground to initially pass a 6-mm screen (Wiley Mill, Thomas Scientific, Swedesboro, NJ), then re-ground to pass a 1-mm screen using a cyclone sample mill (Udy Corp., Fort Collins, CO) for laboratory analysis.

### Detergent Fiber Analysis (DFA)

Hemicellulose and cellulose of stover of 236 RIL were analyzed by modified filter bag technology using ANKOM 2000 instrument (ANKOM Tech., Corp., NY) (Vogel et al., 1999; Wu et al., 2006; Lemus et al., 2008; Anderson et al., 2009). Two modifications were made for neutral detergent fiber (NDF), acid detergent fiber (ADF), and acid detergent lignin (ADL) analyses: a) two empty bags were included in each run as an indicator of bag mass loss during the extraction; one empty bag was placed in the first tray, and the other in last tray to determine the blank bag correction; b) filter bags were dried overnight at 45ºC before to proceeding to next analysis step. Hemicellulose concentration was calculated by subtracting ADF concentration from the NDF concentration. Cellulose concentration was calculated by subtracting ADL concentration from the ADF concentration. Finally, lignin concentration was determined as mass loss following the ashing of the ADF.

### Theoretical Ethanol Calculations

Glucose recovery formulas reported in the literature were inadequate for brown × midrib RIL since they did not account for the advantage of reduced lignin. Theoretical glucose recovery (TGR) from cellulose was estimated by a modified equation (Vogel et al., 2011):

$$\mathrm{TGR}\;(g\text{ k}{\text{g}}^{-1})\;=\;\mathrm{cellulose}\;(g\;kg^{-1})\;\times \;\mathrm{GRE}\;\times \;1.1111$$

where GRE is the glucose recovery efficiency, and 1.1111 is the glucan hydrolysis coefficient (Vogel et al., 2011). The GRE was calculated as follows (Dien et al., 2009):

$$\mathrm{GRE}\;=\;\{[-0.825\;\times \;\mathrm{lignin}\;(g\;kg^{-1})]+\;92.296\}\;÷\;100$$

These modifications adjusted for the differences in lignin concentration among the RIL.

Theoretical xylan recovery (TXR) from hemicellulose was estimated as follows (Anderson et al., 2009; Vogel et al., 2011):

$$\mathrm{TXR}\;(g\;kg^{-1})\;=\;\mathrm{hemicellulose}\;(g\;kg^{-1})\;\times \;1.1353$$

where 1.1353 is the hydrolysis coefficient for xylan.

Theoretical ethanol yield (TETOHY) from cellulosic glucose and hemicellulosic-derived xylan (L ton^-1^) were estimated as follows (Vogel et al., 2011):

$$\mathrm{TETOH}Y_{\left(\mathrm{Cellulose}\right)}=\;\mathrm{TGR}\;(g\;kg^{-1})\;\times \;0.51\;\times \;1.2674\;(\mathrm{ml}\;g^{-1})$$

$$\mathrm{TETOH}Y_{\left(\mathrm{Hemicellulose}\right)}=\;\mathrm{TXR}\;(g\;kg^{-1})\;\times \;0.51\;\times \;1.2674\;(\mathrm{ml}\;g^{-1})$$

where 0.51 is the fermentation coefficient and 1.2674 is the ethanol specific volume. On a unit basis, ml kg^-1^ = L ton^-1^.

TETOHY from soluble sugars (SS) in L ton^-1^ was estimated based on an adjusted version of formulas used by Han et al. and Vogel et al. (Vogel et al., 2011; Han et al., 2013):

$$\begin{array}{c} \mathrm{TETOH}Y_{\left(\mathrm{SS}\right)}=\;[\mathrm{FS}Y\;(\mathrm{kg}\;ha^{-1})\;\times \;\mathrm{Brix}\%\times \;0.90\;\\ \times \;0.51\;\times \;1.2674\;(L\;kg^{-1})]\;÷\;[\mathrm{FS}Y^{*}(\mathrm{ton}\;ha^{-1})] \end{array}$$

where FSY is fresh stover yield, Brix% is concentration of SS in stem juice (Brix value/100), 0.90 is soluble sugars recovery efficiency (sugar yield) and FSY^*^ is fresh stover yield used to estimate L of ethanol per ton. On a unit basis, ml g^-1^ = L kg^-1^.

Therefore, TETOHY (L ton^-1^) from two sources of carbohydrates (structural and SS) was calculated as:

$$\begin{array}{c} \mathrm{TETOHY}\;=\;\mathrm{TETOH}Y_{\left(\mathrm{Cellulose}\right)}\\ +\;\mathrm{TETOH}Y_{\left(\mathrm{Hemicellulose}\right)}+\;\mathrm{TETOH}Y_{\left(\mathrm{SS}\right)} \end{array}$$

Theoretical ethanol production (TETOHP) from cellulosic glucose and hemicellulose-derived xylan (L ha^-1^) were estimated as follows:

$$\mathrm{ETOH}P_{\left(\mathrm{Cellulose}\right)}=\;\mathrm{ETOH}Y_{\left(\mathrm{Cellulose}\right)}\times \;\mathrm{DSY}$$

$$\mathrm{ETOH}P_{\left(\mathrm{Hemicellulose}\right)}=\;(\mathrm{ETOH}Y_{\left(\mathrm{Hemicellulose}\right)}\times \;\mathrm{DSY}$$

where DSY is dry stover yield (ton ha^-1^) in both formulas.

TETOHP from SS (L ha^-1^) was estimated as follows:

$$\begin{array}{c} \mathrm{ETOH}P_{\left(\mathrm{SS}\right)}=\;\mathrm{FSY}\;(kg\;ha^{-1})\;\times \;\mathrm{Brix}\%\;\\ \times \;0.90\;\times \;0.51\;\times \;1.2674\;(L\;{\mathrm{kg}}^{-1}) \end{array}$$

Therefore, TETOHP (L ha^-1^) from two sources of carbohydrates (structural and SS) was calculated as:

$$\mathrm{TETOHP}\;=\;\mathrm{ETOH}P_{\left(\mathrm{Cellulose}\right)}+\;\mathrm{ETOH}P_{\left(\mathrm{Hemicellulose}\right)}+\;\mathrm{ETOH}P_{\left(\mathrm{SS}\right)}$$

### Statistical Analyses

The experimental design for the field evaluation was a randomized complete block with two replications conducted in 2008 and 2009. PROC MIXED procedure (Type III) of SAS 9.3 was used to determine genetic variation and mean differences among RIL. The RIL were considered a fixed effect, Year and Year × RIL interactions were considered random effects. The RIL were assembled into four phenotypic groups to allow a more detailed analysis among contrasting phenotypes. Based on their genetic recombination status (“normal,” “sweet,” “brown,” and “brown-sweet”), the “normal” (non-brown; non-sweet) group was formed by 43 RIL without brown midribs or high stem sugar concentrations (Brix < 12). The “sweet” (non-brown; high stem sugar) group was formed by 108 RIL that carried a mutation for high stem sugar concentration (Brix ≥ 12), but did not have brown midribs. The “brown” (non-sweet; low lignin) group contained those RIL that had brown midribs but were not sweet (10 RIL). The fourth group named “brown-sweet” (recombinants of low-lignin and high stem sugar) were 75 RIL that carried both mutations, one for low lignin (brown midrib) and another for high stem sugar concentration (Brix ≥ 12). We dubbed this group the double mutant group because of the two mutations its members carry. This grouping allowed us to obtain three orthogonal contrasts. The first linear combination compared the double mutant group (“brown-sweet”) against the “normal” RIL group. The second linear combination compared the double mutant group against the “sweet” group. The last linear combination compared the double mutant group against the “brown” group.

### Predictors of Glucose Recovery and Theoretical Ethanol

The PROC REG procedure of SAS 9.3 was used to determine suitable estimators associated with TGR, TETOHY, and TETOHP. The following formula was used to estimate the predictors:

$$Y=\;b_{0}\pm \;b_{1}\times X$$

where X is the explanatory variable and Y is the dependent variable. X was represented by NDF, ADF, cellulose, hemicellulose, lignin (g kg^-1^), SSC (ºBrix), FSY and DSY (ton ha^-1^). The slope of the line is b_1_ and b_0_ is the intercept.

## Results

### Biomass Traits

RIL and Year effects showed significant variation for FSY and DSY. The Year × RIL interaction was only significant for FSY (**Table 2**). The significant variation observed in biomass traits for the Year effect and the Year × RIL interaction effect could be explained by difference on precipitation rates and temperatures between years. Interestingly, the “sweet” and “brown-sweet” RIL groups produced significantly higher FSY than the “normal” and “brown” RIL groups, but not to the parental lines and the control (**Table 3**). The “sweet” RIL group produced significantly higher DSY than the “normal” and “brown” RIL groups, but not to the “brown-sweet” RIL group, the parental lines and the control. This is consistent with the high yield reported in the literature for FSY and DSY in sweet sorghums (Ritter et al., 2008; Murray et al., 2008a). Furthermore, while *bmr* plants (“brown” RIL group) are typically smaller than those of “sweet” RIL, when the *bmr* mutation is combined with the sweet mutation, as in the “brown-sweet” RIL group, the low biomass quantity associated with the *bmr* mutation background effects appear to be compensated for (Rivera-Burgos, 2015).

**Table 2:** Analysis of variance (ANOVA) for biomass compositional traits, and theoretical ethanol traits of brown midrib × sweet sorghum lines over two years.

**Source of variation**	**df**	**Mean square**									
		**FSY**	**DSY**	**SSC**	**Hcell**	**Cell**	**Lignin**	**Lignin** **^+^**	**TGR**	**TETOHY** **^++^**	**TETOHP** **^++^**
Year	1	569**	185*	0.6	128096	774561*	46428*	120856	8904*	102467	3.663*
RIL	235	5.4***	1.7***	26***	703**	1348**	264**	886**	1715**	2316**	0.080**
brown-sweet vs normal	1	123	20*	2649*	79	6203*	25934	87985	129466	272193*	4.343*
brown-sweet vs sweet	1	5.8***	8.8	117	11238	4216	29021	116349	223479	195583*	0.013
brown-sweet vs brown	1	89	18*	723*	1506	4221*	65**	248*	991	12956	2.036**
Year×RIL	235	0.9***	0.5	6.7***	326	613*	67**	205**	500**	667**	0.012**
Error	469	0.6	0.4	4.2	321	487	36	123	265	442	0.009

FSY, fresh stover yield (ton ha^-1^); DSY, dry stover yield (ton ha^-1^); SSC, stem sugar concentration (°Brix); Hcell, hemicellulose concentration (g kg^-1^ dry matter); Cell, cellulose concentration (g kg^-1^ dry matter); Lignin, lignin concentration (g kg^-1^ dry matter); TGR, theoretical glucose recovery (g kg^-1^ dry matter); TETOHY, theoretical ethanol yield (L ton^-1^) and TETOHP, theoretical ethanol production (L ha^-1^). ^+^Lignin concentration g kg^-1^ NDF. ^++^Estimated from structural carbohydrates and soluble sugars. *P-value is less than 0.05, **P-value is less than 0.01 and ***P-value is less than 0.001. Three orthogonal contrasts, each representing phenotypic RIL groups, are shown. The “brown-sweet” RIL group includes ‘double mutants’ combining high stem sugar with low lignin concentration; the “sweet” group is mutant RIL with high stem sugar concentration; the “brown” group is mutant RIL with low lignin concentration; and the “normal” group is non-mutant RIL with low stem sugar and high lignin concentration.

**Table 3:** Mean agronomic (FSY and DSY), compositional (SSC, hemicellulose, cellulose, and lignin), and theoretical ethanol related traits (TGR, TETOHY, and TETOHP) of parental lines (*bmr12* and Brown County), a commercial control genotype *bmr*Atlas, and four phenotypic RIL groups.

Phenotype	FSY (ton ha^-1^)	DSY (ton ha^-1^)	SSC (°Brix)	Hcell^+^ (g kg^-1^)	Cell^+^ (g kg^-1^)	Lignin^+^ (g kg^-1^)	Lignin^++^ (g kg^-1^)	TGR^+++^ (g kg^-1^)	TETOHY^++++^ (L ton^-1^)	TEOHP^++++^ (L ha^-1^)
*bmr*12	72ab*	21ab	10c	163c	261bc	19d	49d	260a	380bc	9264bc
Brown County	75ab	19ab	15a	224b	242c	31bc	75ab	178cd	367cd	11833ab
RIL-normal	65b	18b	11c	243ab	273ab	41a	87a	174d	355d	9430b
RIL-brown	56c	15c	11bc	249a	277a	27cd	61bc	214b	383b	8086c
RIL-sweet	86a	25a	15a	234ab	260c	39ab	84a	172d	370c	14178a
RIL-brown-sweet	83a	23ab	16a	242ab	265bc	26cd	59c	208b	403a	14536a
*bmr*Atlas (control)	69ab	22ab	14ab	235ab	267bc	30bc	68bc	200bc	383bc	12058ab

FSY, fresh stover yield; DSY, dry stover yield; SSC, stem sugar concentration; Hcell, Hemicellulose concentration; Cell, Cellulose concentration; Lignin, Lignin concentration; TGR, theoretical glucose recovery; TETOHY, theoretical ethanol yield and TETOHP, theoretical ethanol production. ^+^Concentration expressed on a dry matter basis. ^++^Concentration expressed on a NDF basis. ^+++^Estimated from cellulose ^++++^Estimated from structural carbohydrates and soluble sugars. *Means in each column followed by the same letter are not statistically different (P < 0.05). The “brown-sweet” RIL group includes “double mutants” combining high stem sugar with low lignin concentration; the “sweet” group is mutant RIL with high stem sugar concentration; the “brown” group is mutant RIL with low lignin concentration; and the “normal” group is non-mutant RIL with low stem sugar and high lignin concentration.

### Stover Carbohydrates and Lignin

RIL effect and the Year × RIL interaction effects showed significant variation for SSC (**Table 2**). The observed variation is explained by phenotypic difference among RIL, and its interaction with precipitation rates and temperatures between years. Not surprisingly, SSC of the “brown-sweet” and “sweet” RIL groups were significantly higher than the other two RIL groups (**Table 3**). However, SSC in these RIL groups were not significantly higher than in Brown County and the control *bmr*Atlas. This high stover quality parameter taken together with the superiority of these RIL groups to the others in measures of stover quantity, all factors in the estimation of theoretical ethanol yield, suggests superior ethanol production from these sorghum lines.

We observed significant variation in Year effect and the Year × RIL interaction effects for cellulose and lignin concentration, but not for hemicellulose concentration. This suggests that hemicellulose concentration is less affected by environmental factors (i.e., precipitation rates, temperatures, et.) than cellulose and lignin (**Tables 1** and **2**). The mean comparison for cellulose showed that the “brown” and the “normal” RIL groups had higher cellulose concentration when compared to the “sweet” RIL group and Brown County, but lower than the “brown-sweet” RIL group, the control, and *bmr12* (**Table 3**). This would be anticipated because as concentrations of one carbon pool (soluble sugars in this case) increase, other carbon pool concentrations must decrease, and cellulose declines were in this case significant. Hemicellulose concentrations were more consistent, and no significant differences were observed among RIL groups (**Table 3**). The most striking difference among the RIL groups was observed in lignin concentration. Here, the *bmr* members of the RIL population (“brown” and “brown-sweet”) showed significantly lower lignin concentration (∼26.5 g kg^-1^) than the other two RIL groups (∼40 g kg^-1^). This represents 60% less lignin concentration in the stover of the *bmr* RIL relative to the others, presumably making the other structural carbohydrates more available to enzymatic hydrolysis (**Table 3**). Based on the negative effect of high concentration of lignin on biomass conversion to ethanol, it is expected that the “brown” and the “brown-sweet” sorghum will yield more ethanol (Dien et al., 2009).

### Theoretical Glucose Recovery, Ethanol Yield, and Ethanol Production

The RIL showed significant differences in TGR from cellulose (**Table 2**). This means that TGR from at least one RIL was significantly higher than the others. Year effect and Year × RIL effects were also significant for TGR. These variations were associated with the effects of precipitation rates and temperatures between years in the RIL performance (**Table 1**). The “brown” and the “brown-sweet” RIL groups showed greater TGR from cellulose than the other two RIL groups (**Table 3**). This supports the hypothesis that lower lignin concentration exposes more structural carbohydrates, like cellulose and hemicellulose, to the process of enzymatic hydrolysis, where they are converted to fermentable sugars. Although TGR levels of the RIL with the *bmr* mutation was not significantly higher than the *bmr* donor, *bmr12*, these RIL showed significantly higher estimates of TGR in comparison to the sweet parent, Brown County and the RIL without the *bmr* mutation (**Table 3**). As expected, *bmr*Atlas showed greater TGR from cellulose than the RIL groups without the *bmr* mutation (“normal” and “sweet”), and was similar to the “brown” and “brown-sweet” RIL groups. Interestingly, *bmr*Atlas and Brown County showed similar TGR from cellulose. This supports our hypothesis that genetic background differences could play a major role in enzymatic hydrolysis of lignocellulosic biomass (**Table 3**).

**Figure 1:**
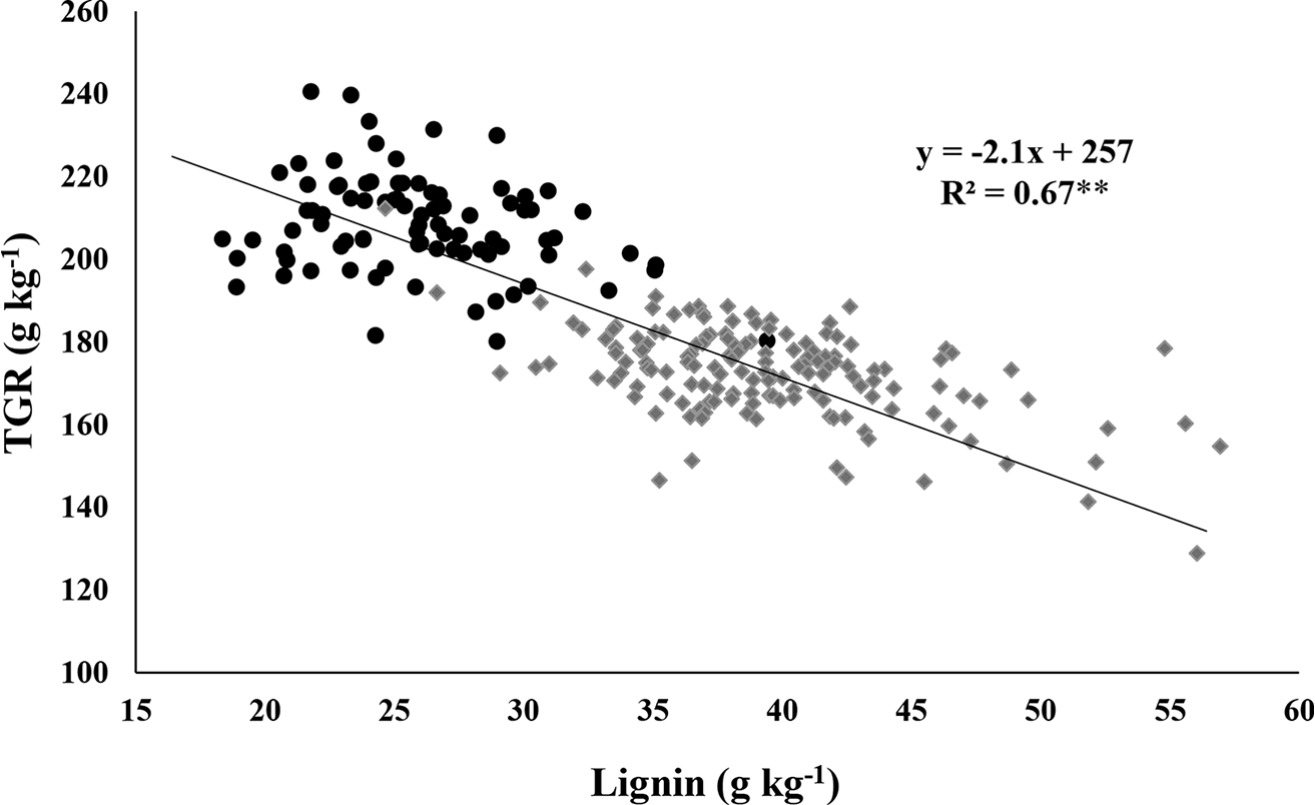
Distribution of theoretical glucose recovery (TGR) from cellulose and lignin measured from stover samples of individual RIL averaged over two years. Data points appearing as black circles represent RIL with brown midribs (“brown” and “brown-sweet” groups), those as grey diamonds are RIL with non-brown midribs (“normal” + “sweet” groups). **Significance at P < 0.01.

The TGR from cellulose of individual RIL of the brown midrib × sweet population plotted against lignin concentration of individual RIL shows that cell wall lignin concentration is negatively associated with TGR (**Figure 1**). Linear regression revealed that 67% of the variability in TGR from cellulose could be explained by stover lignin concentration. The slope of −2.1 indicates that for a reduction of 1 g of lignin per kg of dry matter you can expect TGR to increase by an average of 2.1 g per kg. In this population, 67 RIL, all carrying the brown midrib mutation yielded above 200 g/kg of glucose upon hydrolysis of their lignocellulosic biomass (**Figure 1**). These results portend that this trait improves the quality of lignocellulosic biomass in terms of predicted glucose recovery per unit biomass in this population. Even more, it supports the hypothesis that structural carbohydrates, like cellulose, are more readily available to digestive processes when less lignin is there to bind them (Dien et al., 2009).

**Figure 2:**
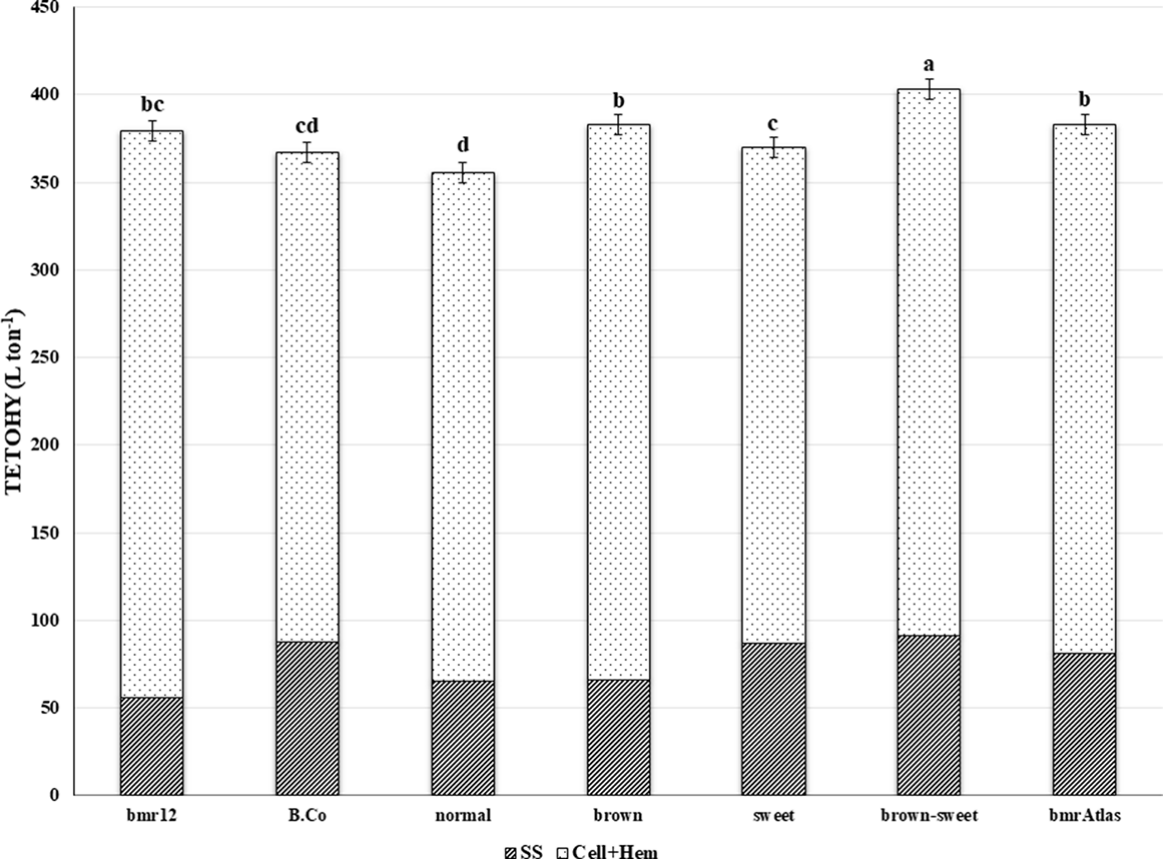
Mean theoretical ethanol yield (TETOHY) from two sources of sorghum stover carbohydrates. Striped bars represent TETOHY from soluble sugars (SS) and the dotted bars represent TETOHY from cellulose and hemicellulose (Cell+Hem) estimates of *bmr* parent (*bmr12*), sweet stem parent (B.Co), four RIL groups (“brown-sweet,” “brown,” “sweet,” and “normal”) and a commercial control (*bmr*Atlas). Bars without the same letter are significantly different (*P* < 0.05). Error bars represent ± one standard error of the mean.

The TETOHY is a function of the amount of structural carbohydrates and non-structural sugars present in stover that is ultimately available for fermentation (**Figure 2**). Significant differences were observed among RIL, indicating that at least one RIL is capable of producing significantly higher amounts of theoretical ethanol than the others, based on stover compositional traits (**Table 2**). The Year × RIL interaction was also significant. This interaction was due to the effects of precipitation rates and temperatures between years on the performance of RIL (**Table 1**). In the mean comparison, the “brown-sweet” RIL group was capable to yielding significant higher amounts of TETOHY than the other three RIL groups (**Figure 2**). The TETOHY mean comparison showed the double mutant RIL group (“brown-sweet”) as first in rank, yielding a significant amount of 403 L ton^-1^ (**Table 3** and **Figure 2**). This high predicted ethanol yield per ton of biomass was possible because sorghums not only have more sugars in their stems, carbohydrates that do not require hydrolysis before being fermented, but they also have increased availability to hydrolysis of the structural carbohydrates, cellulose and hemicellulose, due to reductions in lignin relative to non-*bmr* RIL. Consistent with our hypothesis, we found that combining the high stem sugar concentration and *bmr* mutations into a single line, increased the soluble non-structural carbohydrates (sugars in the stems), thereby maximizing ethanol yields (Murray et al., 2008a; Vogel et al., 2011). No significant differences were observed among TETOHY of the low lignin germplasm material including the “brown” RIL group, *bmr*Atlas and *bmr*12 (genotypes with the *bmr* mutation).

**Figure 3:**
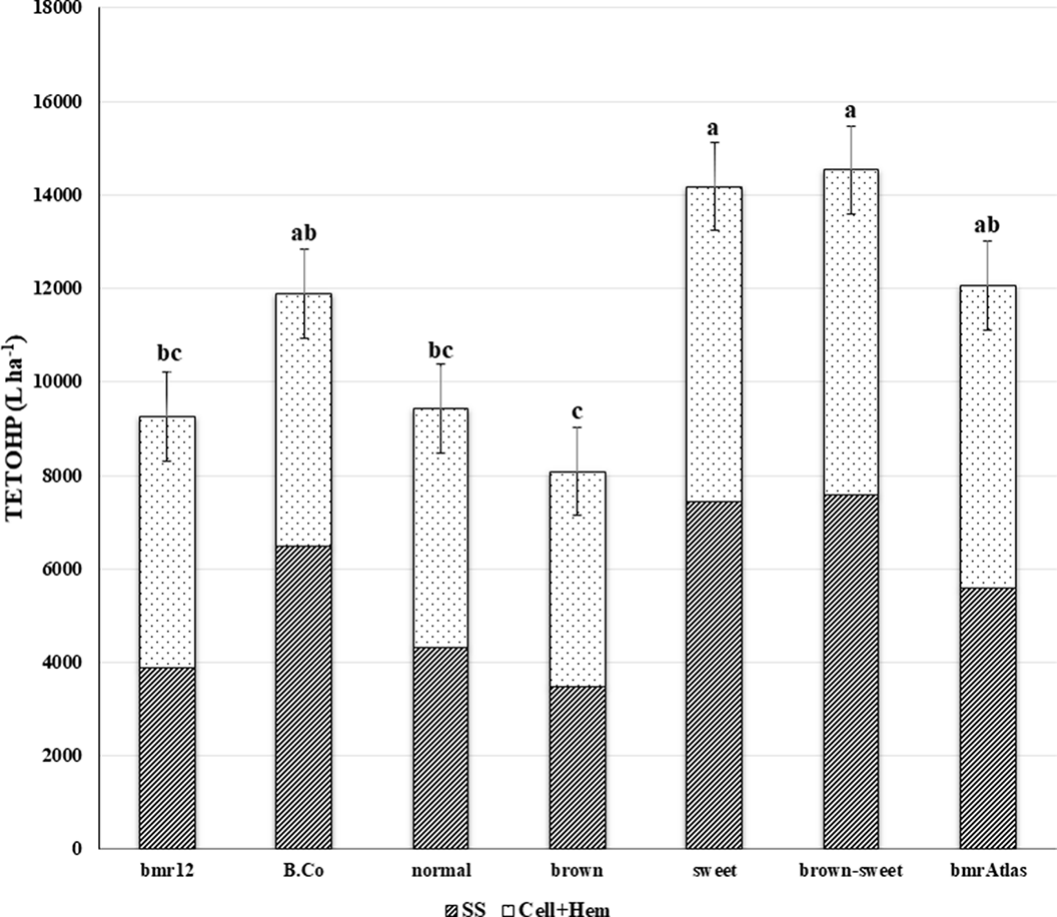
Mean theoretical ethanol production (TETOHP) from two sources of sorghum stover carbohydrates. Striped bars represent TETOHP from soluble sugars (SS) and the dotted bars represent TETOHP from cellulose and hemicellulose (Cell+Hem) estimates of *bmr* parent (*bmr*12), sweet stem parent (Brown County), four RIL groups (“brown-sweet,” “brown,” “sweet,” and “normal”) and a commercial control (*bmr*Atlas). Bars with different letters are significantly different from each other (*P* < 0.05). Error bars represent ± one standard error of the mean.

Similar to TETOHY, ETOHP is also a function of structural and non-structural carbohydrates present in lignocellulosic biomass (**Figure 3**). However, this variable also accounts for biomass productivity (Vogel et al., 2011). In the combined ANOVA of TETOHP there were significant differences among the RIL; and significant Year effects and Year × RIL interaction effects were also observed due to differences in precipitation rates and temperatures between years (**Table 1**). Within RIL, the “brown-sweet” vs. “normal” and “brown-sweet” vs. “brown” were significant. This indicates that the “brown-sweet” RIL group would be expected to produce higher amounts of ethanol than the “normal” and the “brown” RIL groups. The “brown-sweet” RIL group and the “sweet” RIL group were predicted to produce 14,536 and 14,178 L ha^-1^, respectively, and these values were not significantly different from each other (**Table 3**). This is likely much more due to the superior biomass quantity characteristic of the sweet sorghums which tend to be tall plants with thick stems and more leaves than non-sweet sorghums (Rivera-Burgos, 2015). The Brown County parent (with sweet stalk but normal lignin), and the *bmr*Atlas (with low lignin but normal sugar) were predicted to produce 11,883 and 12,058 L ha^-1^ of ethanol, respectively. These amounts were not significantly different from the “brown-sweet” and “sweet” RIL groups. Predicted ethanol production of the “normal” and “brown” RIL groups similar (9,430 and 8,086 L ha^-1^, respectively), and these two RIL groups were not predicted to produce as much ethanol as the “brown-sweet” and the “sweet” RIL groups (**Figure 3**).

### Predictors of Theoretical Glucose Recovery, Ethanol Yield, and Ethanol Production

**Table 4** shows three possible predictors for TGR in the *bmr* × sweet sorghum population as a whole (all 236 RIL). Lignin, hemicellulose and cellulose concentration explained 66%, 11%, and 8% of the variation in glucose recovery, respectively. Most importantly, lignin concentration was negatively correlated with TGR, while hemicellulose and cellulose concentrations were positively correlated. Lignin concentration therefore emerged as best predictor of TGR from cellulose in our *bmr* × sweet sorghum population (Oliver et al., 2005).

**Table 4:** Prediction estimates for theoretical glucose recovery (TGR), theoretical ethanol yield (TETOHY), and theoretical ethanol production (TETOHP) in the entire RIL population as well as the contrasting phenotypic groups.

TGR (*y*)		TETOHY (*y*)		TETOHP (*y*)	
Equation	R^2^	Equation	R^2^	Equation	R^2^
*RIL population*					
*y* = 257 – 2.1 **Lignin**	0.66***	*y* = 448 - 2.0 **Lignin**	0.46***	*y* = -354 + 606 DSY	0.89***
*y* = 65 + 0.5 Hcell	0.11**	*y* = 291 + 6.0 SSC	0.35***	*y* = -1723 + 189 FSY	0.89***
*y* = 102 + 0.3 Cell	0.08*	*y* = 200 + 0.8 Hcell	0.17**	*y* = -1736 + 1082 SSC	0.38***
		*y* = 310 + 0.3 Cell	0.04*		
*“normal” RIL group*					
*y* = 214 - 0.9 **Lignin**	0.32**	*y* = 314 + 3.5 SSC	0.24**	*y* = -855 + 159 FSY	0.84**
		*y* = 406 - 1.3 **Lignin**	0.22**	*y =* 691 + 479 DSY	0.82**
				*y* = 4347 + 481 SSC	0.18**
				*y* = 23252 - 56 **Hcell**	0.10*
*“brown” RIL group*					
*y* = 69 + 0.5 Cell	0.77**	–	–	*y* = -490 + 592 DSY	0.95**
*y* = 79 + 0.4 ADF	0.67**	–	–	*y* = 649 + 139 FSY	0.87**
*y* = 56 + 0.3 NDF	0.54*	–	–		
*“sweet” RIL group*					
*y* = 223 - 1.3 **Lignin**	0.32**	*y =* 177 + 0.8 Hcell	0.47**	*y* = 860 + 548 DSY	0.93**
*y* = 104 + 0.3 Cell	0.15**	*y =* 228 + 0.3 NDF	0.27**	*y* = -869 + 178 FSY	0.88**
*y* = 95 + 0.3 Hcell	0.13**	*y* = 267 + 0.4 Cell	0.21**	*y =* 692 + 949 SSC	0.12**
*y* = 112 + 0.1 NDF	0.08*	*y* = 299 + 4.7 SSC	0.18**		
*y* = 140 + 0.1 ADF	0.04*	*y* = 298 + 0.2 ADF	0.11**		
		*y* = 400 - 0.8 **Lignin**	0.06*		
*“brown-sweet” RIL group*					
*y =* 83 + 0.5 Cell	0.42**	*y* = 193 + 0.4 NDF	0.48**	*y* = -294 + 654 DSY	0.94**
*y* = 117 + 0.3 ADF	0.24**	*y* = 177 + 0.9 Hcell	0.46**	*y* = -311 + 182 FSY	0.91**
*y* = 105 + 0.2 NDF	0.20**	*y* = 234 + 0.6 Cell	0.45**	*y* = -4547 + 1259 SSC	0.24**
*y* = 234 - 1.0 **Lignin**	0.12*	*y =* 255 + 0.5 ADF	0.37**		
*y* = 140 + 0.3 Hcell	0.07*	*y* = 310 + 5.9 SSC	0.34**		

NDF, neutral detergent fiber (g kg^-1^ dry matter); ADF, acid detergent fiber (g kg^-1^ dry matter); SSC, stem sugar concentration (°Brix); Hcell, hemicellulose concentration (g kg^-1^ dry matter); Cell, cellulose concentration (g kg^-1^ dry matter); Lignin, lignin concentration (g kg^-1^ dry matter) and FSY, fresh stover yield (ton ha^-1^); DSY, dry stover yield (ton ha^-1^). In bold, negatively correlated variables. ***Significance at P < 0.001; **significance at P < 0.01; *significance at P < 0.05.

when the same analysis was applied to the RIL grouped according to whether or not they carried the two quality mutations (**Table 4**), the predictive power of other components for glucose recovery became apparent. Within the “brown-sweet” and “brown” RIL groups, cellulose explained 42% and 77% of the total variation in TGR from cellulose, respectively. Lignin concentration within these groups, of course, did not vary greatly since they all contained the *bmr* mutation and so all had generally reduced lignin concentration with respect the non-*bmr* members of the population. Therefore, the contributions of the other predictors in these two groups are unmasked. The ADF concentration, that includes both lignin and cellulose, and the NDF concentration, that includes cellulose, hemicellulose and lignin, also explained some of the variation in TGR from cellulose. For the “brown-sweet” RIL group, ADF and NDF concentrations explained 24% and 20% of the variation in TGR from cellulose respectively. For “brown” RIL group, ADF and NDF concentrations explained 67% and 54%, respectively. Within the “sweet” and “normal” RIL groups, lignin concentration explained 32% of the variation in TGR from cellulose in both groups. This reflects the presence of background variation in lignin concentration among “normal” lines not carrying the *bmr* mutation, although, this variation was not as great as when comparing to the *bmr* lines that carry a mutation for low lignin concentration. The variation in lignin concentration within these groups was enough, however, to show even here that lignin concentration is an excellent predictor of TGR from cellulose, the only significant one within the “normal” RIL group and the major one within the “sweet” RIL group. In the latter group, cellulose and hemicellulose were also highly significant predictors of TGR from cellulose at 15% and 13%, respectively, with less significant determinants being NDF (8%) and ADF (4%) (Dien et al., 2006; Dien et al., 2009).

The TETOHY shows a slightly different trend when compared to TGR, though lignin (46%) still emerged as a major predictor in the RIL population (**Table 4**). Included here, SSC (35%) representing the contribution of soluble carbohydrates, also emerges as a major predictor of TETOHY over the entire population (**Table 4**).

when the linear relationships between lignocellulosic biomass components are considered within each RIL group (**Table 4**), other determinants become apparent. Since both lignin concentration and SSC are co-confounded in the “brown-sweet” RIL group, that is, all member lines having relatively low lignin concentration and a high SSC, many suitable predictors were observed. The concentrations of NDF, hemicellulose, cellulose, and ADF explained 48%, 46%, 45%, and 37% of the variation in TETOHY in this group, respectively. Interestingly, even SSC explained 34% of the variation in ethanol yield, reflecting the high variation of Brix measurements among these “brown-sweet” lines grouped here because their SSC exceeded 12ºBrix. This reflects the more complex genetics of the sweet mutation compared to that of the *bmr* mutation. Within the “sweet” and “normal” RIL groups, SSC (18% and 24%, respectively) were significant predictors of TETOHY. Here again, there was enough variation among the members lines in Brix measurements to see associations with ethanol yield. This was also true for lignin concentration, even though neither group contained individuals with brown midribs (Dien et al., 2009).

Ethanol production is highly dependent on the quantity of biomass that is used as feedstock. It was observed significantly positive linear associations of TETOHP with DSY, FSY and SSC for all 236 RIL of the *bmr* × sweet sorghum population (**Table 4**). DSY and FSY, explained most of the total variation for TETOHP, each accounting for 89%. This means that biomass quantity is the most important determinant of ethanol production. There is also a strong association, though less than half of the stover yield measures, of the biomass quality factor, SSC which explained 38% of TETOHP variation over the entire population.

The biomass quantity parameters (DSY and FSY) were also the major predictors of TETOHP when the population was analyzed in groups based on presence or absence of the sweet and bmr mutations. In all groups, these two quantity measures predicted 82% to 95% of the TETOHP. In all but the “brown” RIL group, variation in SSC was significantly and positively correlated with TETOHP. This also support the hypothesis that biomass quantity traits, FSY and DSY, are the major determinant for ethanol production (**Table 4**) (Vogel et al., 2011; Han et al., 2013).

## Discussion

Biomass conversion is a key process required to produce ethanol as source of renewable energy. Over the last decade, the industrial sector has focused on improving this process by designing new methodologies to efficiently hydrolyze and ferment lignocellulosic biomass (Wu et al., 2006; Lemus et al., 2008). However, to reach significant bioconversion efficiency, products such as sulfuric acid and genetically engineered microbes capable of breaking-down structural carbohydrates to fermentable sugars are required in large amounts (Anderson et al., 2009). Genetically enhanced germplasm with high biomass yield and enhanced compositional quality have the potential to improve sorghum stover conversion to ethanol; attributes recently shown to enhance both the economic and environmental performance of currently available biomass-to-ethanol conversion systems (Torres et al., 2016). The ability of the *COMT* gene mutation to reduce lignin concentration in lignocellulosic biomass showed positive effects towards the improvement of biomass conversion efficiency of the *bmr* lines in this study, at the population level, consistent with previous studies (Ohgren et al., 2007; Ritter et al., 2008; Vogler et al., 2009).

The *bmr* mutation makes stover carbohydrates readily fermentable (Ritter et al., 2008; Vogler et al., 2009); however, it generally reduces plant fitness, resulting in shorter plants, delayed maturity, and increased tendency to lodge (Pedersen et al., 2005). When both mutations are combined, as in the RIL of the “brown-sweet” group, the reduced fitness caused by the *bmr* mutation is compensated for by the introduction of the sweet mutation, with some individuals even exceeding the performance of RIL carrying only one of the mutations (Rivera-Burgos, 2015). Evidence in this study suggest that the sweet mutation not only improves the SSC quality trait, but the stover yield of *bmr*, high sugar recombinant sorghum lines is increased, boosting theoretical ethanol production. This is especially noteworthy because most recent studies (Marsalis et al., 2010; Gill et al., 2014; Guragain et al., 2017; Kumar et al., 2018) confirm those reported previously (Oliver et al., 2004; Pedersen et al., 2005) regarding the inferior and/or variable agronomic performance of most sorghum lines with the *bmr* trait. This shortcoming has slowed deployment of this trait in both the forage and bioenergy sectors of agriculture.

Enhanced ethanol yields would be expected from *bmr* sorghums because their reduced lignin content exposes the structural carbohydrates to the processes of hydrolysis that break the polymers cellulose and hemicellulose into easily fermentable residues (Badger, 2002; Dien et al., 2009; Murray et al., 2009). On the other hand, sweet sorghums, by virtue of having more ready-to-ferment sugars already present at increased amounts, at least in the stem portion of the stover, would have higher TETOH relative to the “normal” RIL group. In this study, both biomass quality mutations (sweet, and low lignin) do give significantly higher TETOHY than the sorghum lines without either mutation (“normal” RIL, 355L ton^-1^), but the “brown” RIL group (383L ton^-1^) more so than the “sweet” RIL group (370L ton^-1^). This is perhaps not too surprising considering the effects of each mutation on overall availability of fermentable carbohydrates in the plant (Bout and Vermerris, 2003; Rooney et al., 2007; Murray et al., 2009). While the sweet mutation causes more sugars to accumulate, carbohydrates which are immediately available to fermentation, this accumulation only occurs in one part of the plant, the stem. The *bmr* mutation affects sorghum stover, the reduced lignin exposing the greater structural carbohydrates, cellulose and hemicellulose, components of every cell wall, to the processes of hydrolysis (Porter et al., 1978; Saballos et al., 2008). While cellulose and hemicellulose require an extra step before fermentation can occur, these polymers are present in higher amounts than soluble sugars of sorghum stover, that the ethanol yield of the overall process is enhanced more by the widespread expression of the *bmr* mutation and lower lignin throughout all tissues of the plant (Prasad et al., 2007; Zhao et al., 2012). The “brown” RIL group yielded significantly more theoretical ethanol than the “sweet” and the “normal” RIL groups, and the “sweet” RIL group was capable of yielding significantly higher theoretical ethanol than the “normal” RIL group (383, 370, and 355 L ton^-1^, respectively). When single mutations for lignocellulosic biomass enhancement were compared independently, the low lignin *bmr* mutation had a more significant effect on predicted ethanol yield than stem sugar “sweet” mutation, a result also previously reported in other studies (Badger, 2002; Dien et al., 2009; Masarin et al., 2011).

Based on data from this study, biomass quantity emerges as the most important factor in determining ethanol *production*. Traits that contribute to plant size, such as tall leafy plants with thicker stems that contribute to production of more total biomass per area increase ethanol production. Biomass quality traits, like the *bmr* mutation that exposes structural carbohydrates to hydrolysis, or the sweet mutation that increases the ready-to-ferment sugar concentration of the raw plants, as well as those that yield more ethanol per unit biomass, contribute to feedstock improvement at the level of ethanol *yield*. In addition to enhancing the extent of cell wall degradation, the *bmr* trait has been shown previously to accelerate the rate of cell wall digestion (Cherney et al., 1985; Cherney et al., 1986). This may further increase the practical value of the *bmr* trait by increasing the throughput of feedstock in a bio-ethanol production facility. From a breeding perspective, initial selection for biomass quantity traits would tend to contribute to improved ethanol productivity the most. However, as one reaches the upper limits to yield potential (i.e., genetic variability, genetic stability, land availability, single cropping season, etc.) for a crop like sorghum, genetic changes in quality traits that improve the efficiency by which the biomass is converted to ethanol become important.

Compared to other lignocellulosic and stem juice bioenergy crops, that the *bmr*-sweet sorghum lignocellulosic biomass give much higher amount of ethanol production is significantly important. *Miscanthus*, sugar beet (*Beta vulgaris*), maize (*Zea mays*), rice (*Oryza sativa*), wheat (*Triticum aestivum*), sugarcane (*Saccharum officinarum*), sweet and forage sorghum (*Sorghum bicolor*) produce no more than 6,500 L of ethanol per ha (Gupta and Verma, 2015). This ethanol is produced from both structural carbohydrates (bagasse) or soluble carbohydrates (stem juice) (Anderson et al., 2009; Nelson et al., 2011). The efficient utilization of two sources of carbohydrates to produce ethanol from this genetically improved lignocellulosic biomass offers an attractive added value to farmers and industry (Badger, 2002; Masarin et al., 2011). The results of this study showed evidence of the importance of the *bmr* and sweet sorghum lignocellulosic biomass quality and quantity factors influencing ethanol production at an industrial scale (Moller, 2005; Wu, 2008; Wang and Zhu, 2010).

In summary, large-scale production of a genetically enhanced lignocellulosic biomass would help to improve bioconversion efficiency required by the bio-refineries. Traits that enhance biomass quality such as the low lignin and high stem sugar concentrations, as well as traits contributing to increased biomass quantity per unit land (i.e., FSY and DSY) can improve ethanol production, driving down cost without harmful environmental effects. In this study, we found that biomass quantity traits were the most important determinants of ethanol production, and biomass quality traits enhanced sorghum biomass conversion. The genetically enhanced sorghum biomass (*bmr* × sweet sorghum) offered two sources of stover carbohydrates (soluble carbohydrates and structural carbohydrates) to significantly increase ethanol yields.

## Author Contributions

LR-B designed and conducted the research, collated and analyzed the data, and produced the manuscript draft. GE developed the sorghum mapping population and suggested the research question. He also supervised the research and edited the manuscript. JV supervised the research and edited the manuscript.

## Funding

This research did not receive any specific grant from funding agencies in the public, commercial, or not-for-profit sectors. LR-B’s graduate research support was covered by GE’s Distinguished Professor research allowance from Purdue University.

## Conflict of Interest Statement

The authors declare that the research was conducted in the absence of any commercial or financial relationships that could be construed as a potential conflict of interest.
